# The Impact of Voice Leading and Harmony on Musical Expectancy

**DOI:** 10.1038/s41598-020-61645-4

**Published:** 2020-04-03

**Authors:** Leona Wall, Robert Lieck, Markus Neuwirth, Martin Rohrmeier

**Affiliations:** Digital and Cognitive Musicology Laboratory, École Polytechnique Fédérale de Lausanne, 1015 Lausanne, Switzerland

**Keywords:** Human behaviour, Perception

## Abstract

In Western tonal music, voice leading (VL) and harmony are two central concepts influencing whether a musical sequence is perceived as well-formed. However, experimental studies have primarily focused on the effect of harmony on the cognitive processing of polyphonic music. The additional effect of VL remains unknown, despite music theory suggesting VL to be tightly connected to harmony. Therefore, the aim of this study was to investigate and compare the effects of both VL and harmony on listener expectations. Using a priming paradigm and a choice reaction time task, participants (N = 34) were asked to indicate whether the final chord in a sequence had a different timbre than the preceding ones (cover task), with the experimental conditions being *good* and *poor* VL or harmony, respectively. An analysis with generalised mixed effects models revealed a significant influence of both VL and harmony on reaction times (RTs). Moreover, pairwise comparison showed significantly faster RTs when VL was *good* as compared to both VL and harmony being *poor*, which was not the case when only harmony was *good*. This study thus provides evidence for the additional importance of VL for the processing of Western polyphonic music.

## Introduction

One core issue of music cognition concerns the processing of chord sequences and their underlying syntax^1,2^, which in Western tonal music consists of two central components: voice leading (VL) and harmony. Music theory strongly emphasizes the close intertwinement of VL and harmony in common-practice tonality (approx. 1650–1900)^3^. However, experimental studies on expectancy in polyphonic music have primarily focused on harmony^4–6^, sidelining the additional impact of VL. Based on the number of studies on harmonic expectancy, there seems to be a broad consensus among music psychologists that the perception of chord sequences from polyphonic music is primarily determined by harmony^4^. Therefore, the additional effect of VL in polyphonic music remains unknown. The primary goal of this study is to investigate the role of VL in expectation formation as well as the interaction between VL and harmony.

In music theory, VL concerns the treatment of tones belonging to individual voices with respect to their horizontal and vertical (intervallic) dimensions; in contrast, harmony deals with chords (that is, combinations of tones in multiple voices) and the transition between them. In traditional accounts of tonal harmony, chords are described as stacks-of-thirds based on a root note; the functional relationships between chords (e.g., dominant-to-tonic) are primarily assessed on the basis of the relationships of their roots^7,8^. In terms of syntactic coherence, it is assumed that all chords in a sequence could in principle be converted to their root-position; the primary role of chord inversions (i.e., chords in which the root note is not the lowest note) is to allow for smooth VL in the bass^8^.

In studies of music cognition, expectancy offers a useful empirical tool since expectations are indicative of how musical structures are processed^9^. For testing listening expectations, a priming paradigm with a choice reaction time task can be used^5,10^. A priming effect can be observed when a specific stimulus influences the processing of a subsequent target stimulus, which becomes apparent through different reaction times (RTs) for different degrees of expectation^9^. Faster RTs indicate a mental anticipation of the target sonority and therefore a facilitated processing; in contrast, surprising events lead to slower RTs^11,12^.

One important prior work on harmonic priming includes a study which found a significant effect of VL on participants’ performances in a RT paradigm^13^. In this study, two types of target chords were used, a tonic chord and a subdominant chord, the latter being expected to lead to less accurate and slower responses after a dominant prime. As a novel insight, the size of the priming effect was found to depend on the correctness of VL (good vs. bad), with faster responses in the correct VL condition. However, that study investigated the effect of only one type of VL (parallel VL of perfect vertical intervals, i.e. unisons, fifths, and octaves) on harmonic priming, while the effect of VL alone remains unknown.

The present study aims to investigate the role of VL and harmony in more detail by quantifying their respective influence on listener expectations. Using a priming paradigm, priming sequences were followed by targets that fulfilled four different combinations of *good* and *poor* VL and harmony, respectively (V+H−, V−H+, V+H+, and V−H−). A timbre discrimination task was introduced as a cover task to measure RT. The V+H+ condition was expected to yield the fastest RTs, assuming that VL and harmony need to be coordinated to fulfill listeners’ expectations; in contrast, the V−H− condition was expected to lead to the longest RTs. The key interest lay in the comparison of RTs for targets with either VL or harmony being *good*, and the other *poor*. By using this experimental setup, the cognitive load and the degree of mental anticipation reveal participants’ expectancy to better understand the mechanisms involved in the processing of musical syntax.

## Materials and Methods

**Figure 1:**
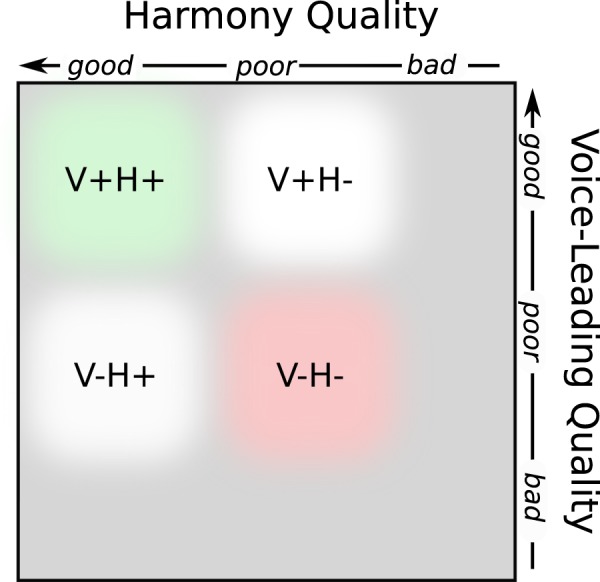
Design of the targets. Each prime sequence ends with a target chord that corresponds to the different experimental conditions. These conditions are *good* and *poor* voice leading or harmony, respectively, as well as control conditions (both *good* and both *poor*, respectively). The stimuli were designed in such a way that neither VL nor harmony were extremely dysfunctional (*bad*) – such as four (chord) tones randomly distributed over a given pitch range – but only moderately *poor* in the respective conditions.

The experiment was designed as a choice RT task based on a priming paradigm. Each prime-target combination was rendered with a piano sound using two different timbres (“normal” and “bright”) for the target condition. The cover task was to discriminate the different piano timbres and RTs were measured. The targets corresponded to *good* and *poor* VL and harmony, respectively (V+H−, V−H+) as well as control conditions (both *good* or both *poor*, V+H+, V−H−, respectively; see Fig. 1). The present study was carried out in accordance with Swiss and EU guidelines and regulations, and was approved by the institutional Human Research Ethics Committee under the number 027–2018.

### Participants

Thirty-six participants were recruited through poster announcements at the institution’s campus and personal recommendation of participants among each other. Written informed consent was obtained before the study, and participants received monetary reimbursement after completion. To assess musical sophistication, participants were asked to fill in the Gold-MSI questionnaire^14^.

### Experimental procedure and material

The experimental set up employed a browser-based interface coded in Python 3 on a Django Server. Participants were seated in a quiet room in front of a computer screen. Instructions were given by the same person in each experiment. Two sequences were played to provide examples of the sequences before the practice phase: one sequence with a V+H+ condition and one sequence with a V−H− condition, both first with the normal and then with the bright timbre. All sequences were presented via headphones (beyerdynamic DT-990 PRO (250 Ohm)).

The experiment consisted of three parts: a practice phase, an experimental phase, and a phase of answering the Gold-MSI questionnaire. The task in both the practice and the experimental phases was to discriminate between the two piano timbres in the last chord of a 9-chord sequence. The timbre of the target was either the same or different from the “normal” timbre of the prime sequence (the question was “Did the piano change in the last chord? Decide as fast as possible”).

A progress bar appeared on the screen to indicate the progress of the sequence as a visual aid. Also, two boxes (“F” and “J”) reminded the participants which button on the computer keyboard indicates which decision (keys were randomized for each participant). All sequences were separated by a white noise of 5 seconds, and participants were able to proceed to the next sequence when pressing the spacebar. The sequences were played in random order, and sequences used in the practice phase were not reused in the experimental phase in order to avoid a learning (memorisation) effect.

The practice phase consisted of 14 sequences (six V+H+ conditions and one V−H− condition, each played with normal and bright timbre targets, in random order). After each sequence, participants received feedback on the screen indicating whether the answer was correct or incorrect. They had to score at least 75% correct answers and feel confident to do the task in order to be considered for the experimental phase. Participants who did not reach the score were allowed to continue practising until they reached the 75% score.

Thus, the practice phase served multiple purposes. First, it ensured participants would reliably hear the timbre difference and hence be able to accomplish the cover task. Also, participants were familiarised with the task in order to avoid significant learning effects during the experimental phase. Additionally, the types of stimuli and the musical style (that of common-practice tonal harmony) were introduced, therefore predominantly stimuli with a V+H+ condition were used in the practice phase. However, one stimulus (played with each timbre for the target) with a V−H− condition was included to minimise surprise for those stimuli in the experimental phase.

The experimental phase consisted of 64 sequences (8 (prime) × 4 (target) × 2 (timbre)). It was set up in the same way as the practice phase, except for the fact that no feedback was provided. After completion of the experimental phase, participants filled in the Gold-MSI questionnaire.

### Musical stimuli

**Figure 2:**
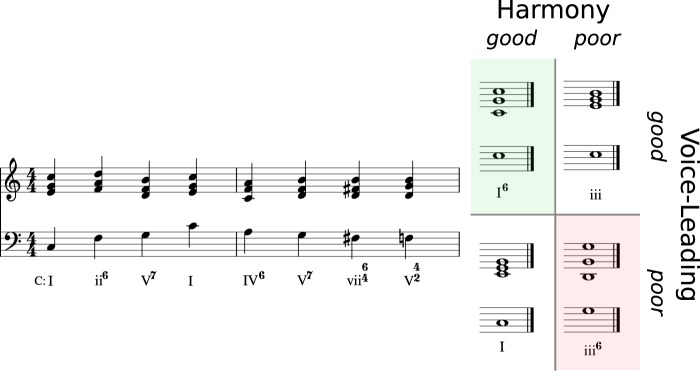
An example stimulus consisting of a prime sequence and four different targets (V+H+, V+H−, V−H+ and V−H−).

The stimuli were designed to test the extent to which listener expectations are guided by VL, harmony, or a combination of both. Therefore, eight different prime sequences of eight chords each were composed, which were followed by four different target chords each (see Fig. 2 for an example stimulus and Supplementary Material for all scores and audio files). The targets were designed to end the sequences in ways corresponding to the experimental conditions, namely *good* VL but *poor* harmony (V+H−), vice versa (V−H+), and with both conditions being *good* (V+H+) or *poor* (V−H−). In each stimulus, the first four or five chords served to unequivocally define a tonal reference point by means of common closing patterns (i.e., cadences).

For the domain of harmony, the key criterion is *root motion*^7,15^. In common-practice tonality, there is a strong preference for root motion to be in descending fifths (this also applies to the Jazz idiom^16^), followed by descending thirds and ascending seconds; root motions inverting these interval directions (ascending fifth, ascending third, and descending second) are less prominent^17–19^. This distribution sets common-practice tonality apart from Pop/Rock tonality, which is characterised by almost a reversal of these frequencies^20^.

Root motion is a necessary condition for the emergence of functional relationships between harmonies, with descending-fifths relationships defining local dominant-to-tonic progressions^7,21^. Chords belonging to the same octatonic pitch collection (chords that are minor thirds, or stacks thereof, apart) were treated as functionally equivalent^22^, which accounts, for instance, for the “tritone substitution” well-known from Jazz but also applicable to some situations in common-practice tonality^3^. There are some recent attempts at formalising and implementing the rules of tonal harmony that we are drawing on in this paper^1,23,24^.

Note that chords are denoted here by Roman Numerals, following a widespread international convention (see Supplementary Material). Uppercase numerals refer to major-mode chords, lowercase numerals denote minor-mode chords. We examine three chord types and their transitions: dominants (V or V^7^ and their inversions, global and local) and two types of predominants: subdominants (IV and ii) and augmented sixth chords. The “good” harmony condition is designed to conform to the following rules:

- We expect dominants to resolve to their tonics (global or local), which is the case in Stimuli 2, 4 and 7 (globally) as well as Stimuli 3, 6 and 8 (locally).
- Further, we expect subdominants (acting as predominant functions) to proceed to their dominants (see Stimulus 5).
- Finally, we expect an augmented sixth chord to act as a predominant and hence to resolve to the dominant (see Stimulus 1).

The condition of “poor” harmony results from denying these transition conventions:

- Dominants *not* resolving to their tonics, but
  - to global iii (Stimulus 2) or local iii (Stimuli 6 and 8)
  - to VI (Stimulus 4), which is less expected than the tonic (in the minor mode)
  - to iv (Stimulus 7) or IV (Stimulus 7).
  - Subdominants (predominant) *not* proceeding to dominant but to iii (in major; Stimulus 5).
  - Augmented sixth chords (as a more local predominant) *not* resolving to dominant but to III (in minor), where III is a major third apart from the dominant and hence does not act as a dominant substitute.

Unlike the harmonic domain, the voice-leading domain involves both vertical and horizontal intervals. Sonorities (and sequences thereof) are defined by the intervallic relationships of their composite tones, rather than by chordal roots. Based on Aldwell and Schachter^15^ and Gauldin^3^, as well as (experimental) work on expectancy^25,26^, we draw on the following heuristic criteria which are necessary to obtain *good* VL.

First, there are two rules of minimal voice leading, involving (1) a preference for sustaining notes shared by adjacent sonorites, and (2) a preference for small steps over leaps^27,28^. Rule (3) governs the treatment of dissonances and leading-tones (scale degree 7 in the major and minor modes), which both are expected to display (stepwise) resolution. Another set of rules is based on melodic and intervallic inertia: (4) there is a preference for continuing a given melodic pattern over deviating from it^29–31^; (5) there is a preference for retaining an outer-voice intervallic pattern (e.g., chains of parallel 3ds, 10ths, 6ths, etc.). There are two rules that enable the perception of independent voices: (6) avoidance of parallel perfect intervals (unisons, fifths, or octaves); and (7) preference for contrary motion. In general, when these rules and the preferences they express conflict with one another, we designed the stimuli such that a maximum number of rules have been satisfied.

Furthermore, we did not systematically manipulate whether the stimuli were tonally closed or open, that is, whether they ended in the same key in which they began. The same applies to the final melodic interval (size and direction) of the soprano voice, and whether the targets were chromatic or belonged to the Rock/Pop idiom. These factors were added as additional (post-hoc) variables to examine possible confounds. Finally, care was taken that effects of sensory memory were limited (e.g.^4^).

The sequences were composed by a music theory expert (MN) and evaluated by members of the research group prior to the experiment to ensure the functional quality of the stimuli (details can be found in the Supplementary Material).

The tempo was set to 80 beats per minute. The scores were converted into MIDI (MuseScore 2.3.2) and then rendered and exported as .wav files (Pianoteq PRO 6.3.0). The priming sequences were rendered using a “normal” timbre, and each target was rendered once with the same timbre as the priming sequence, and once with a different timbre (“bright”). For the normal timbre, the presets for the acoustic piano “Steinway D Home” were used. For the bright timbre, settings for this preset were modified: voicing (hammer hardness for piano, mezzo and forte) was set to the maximum value (2.0).

### Data analysis and statistical procedures

Data analysis was performed using RStudio (Version 1.1.463), with packages plyr, ggplot2, lme4, lmerTest and emmeans.

Data of participants who did not meet the inclusion criterion (minimum 75% success rate in the practice phase) were excluded from analysis. Also, RTs ≤ 100 ms were regarded as accidentally premature answers and the corresponding data points were removed. Outliers were calculated for each target separately (>mean + 2 SD) and removed, along with incorrect answers. Where equal numbers of observations were needed, the RTs closest to the median in the variables with more observations were removed. Data from the “bright” timbre, which were generated due to the cover task, were also excluded, because the change in timbre affects the perceived melodic interval in the top voice between the final prime chord and the target chord, thus rendering the conditions to test impossible to predict^32,33^.

RT data was tested for normal distribution by visual inspection and by using the Shapiro-Wilk test. In a first step, the Wilcoxon signed rank test was used to compare the RTs of different conditions. Afterwards, a generalised mixed effects model (glmer) with the optimizer “Bobyqa” was used to analyse the RT data, and the gamma distribution was chosen to approximate the underlying distribution, as previously suggested^34^. First, variables that could improve the model were identified: the null model contained a random effect for target, and in the subsequent models, different variables were added and the model fits were compared using likelihood ratio tests. Variables that significantly improved the null model were used in the next step, where target was the fixed effect and the variables identified were successively added as random effects. The model with the best fit (according to the likelihood ratio test) was then taken for a pairwise comparison with “emmeans” of the four different conditions with Tukey correction for multiple comparisons.

A second model was fitted to the data, which contained VL and harmony with effect-coded variables as fixed effects including their interaction and random effects as mentioned above. On this model, an ANOVA was performed. Results were regarded as significant when P < 0.05.

## Results

### Overview

Out of the 36 participants, 34 reached the required success rate for timbre discrimination in the practice phase and were therefore included in the analysis. The participants were 28 ± 6 years old (mean ± SD; range: 18–40). Their general musical sophistication according to the Gold-MSI questionnaire was 68 ± 17 (mean ± SD; range: 32–100).

**Figure 3:**
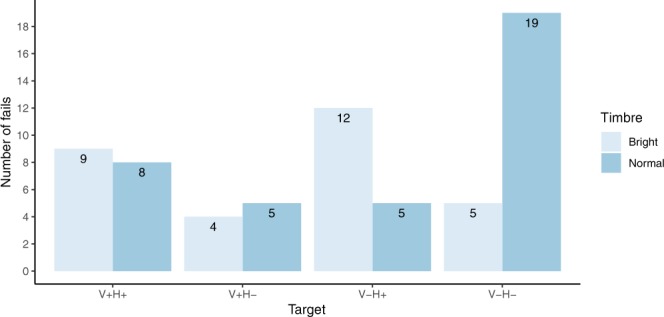
Number of incorrect answers per target (N = 272 per target and timbre, except for V−H+, where N = 271 for each timbre).

The overall success rate including the stimuli with the “bright” target was 96.6%. The number of fails (incorrect answers) was highest in the stimuli with a normal timbre in the V−H− condition (Fig. 3).

**Figure 4:**
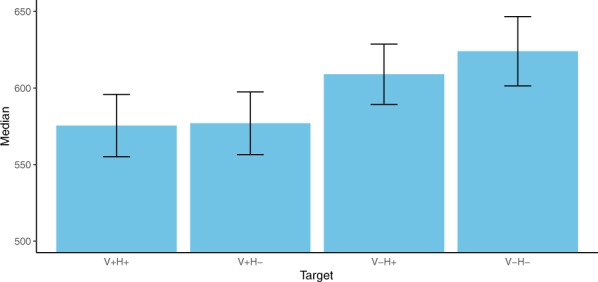
Median reaction time and SE for the timbre discrimination task for each target.

One observation was removed from the dataset as premature answer, and 37 answers were removed because they were incorrect. 5.3% of the data (56 of 1050 observations) was then removed because RTs exceeded the mean + 2 SD (cutoff V+H+: 1312 ms, V−H+: 1320 ms; V+H−: 1349 ms; V−H−: 1430 ms). In the resulting dataset, RT was shortest (median ± SE: 560 ± 13 ms) in the V+H+ condition and longest (606 ± 16 ms) in the V−H−condition. Notably, RT for the V+H− condition was 566 ± 13 ms, and 591 ± 12 ms in the V−H+ condition (Fig. 4). The Shapiro-Wilk test suggested that the data were not normally distributed. The effect of musicality on participants’ reaction times did not seem to follow a trend and also the “final melodic interval” did not seem to have a major impact on RT (see Supplementary Material).

**Figure 5:**
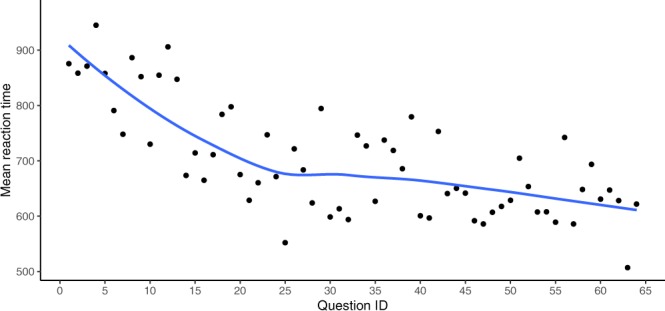
Median reaction time per question ID.

When plotting the mean RT against stimulus order, RT was clearly decreasing over the course of the experiment (Fig. 5). As the stimuli were played in randomised order for each participant, this trend affects all conditions equally. Still, stimulus order was included as a random effect in the generalised mixed effects model (see below).

### Wilcoxon signed rank test

As the number of observations was not equal for the four conditions, 30 observations in total were removed from the dataset to perform the Wilcoxon signed rank test (12 in the V+H− condition as well as 9 in the V−H+ condition and the V+H+ condition each, resulting in N = 241 observations per condition). The Wilcoxon signed rank test yielded a significant difference of RT when VL was *good* (V+H− and V+H+) vs if VL was *poor*, (V−H+ and V−H−; P = 0.005). Also, there was a significant difference when harmony was *good* (V−H+ and V+H+) vs *poor* (V−H+ and V−H−; P = 0.04).

### Generalised linear mixed effects models

In the first step, a null model was fitted to the data with the target as a random effect. “Stimulus order” and “participant” as additional random factors improved the model significantly. They were therefore included in the final models, along with the hierarchical information on the targets nested within primes. Confirming our visual inspection of the data, the variable “final melodic interval” did not significantly improve the model. Similarly, taking into account the stylistic provenance of prime and target, in particular whether they occurred often in Rock/Pop idioms (see Musical Stimuli), did not improve the model significantly. The same is true for whether or not the target was chromatic and whether it led to closure or remained open. General sophistication (“musicality”) improved the model, but when it was added as nested in participants, the model was not significantly better than the one with only participant as a random variable.

In the next step, “target” was set as fixed and “participant” as random effect (AIC 12,837). In the subsequent two models, “stimulus order” (AIC 12,792) and stimulus order along with the hierarchical information (AIC 12,778) were added, which both significantly improved the model fit (P < 0.001) according to the likelihood ratio test.

Therefore, the model used for further analysis contained “target” as a fixed effect, and “participant”, “stimulus order” and hierarchical information as random effects. The Q-Q plot of the model’s residuals indicated a fairly good fit of the model to the data (Supplementary Material Fig. F11).

Using the “emmeans” package, the model was then used for a pairwise comparison of the different targets. The results are shown in Table 1.

**Table 1:** Results of the multiple comparisons of reaction times for different conditions by the generalised linear mixed effects model with target as a fixed effect. P-values are Tukey corrected.

Comparison	Estimated difference	Standard error	P-value (corrected)
V−H−/V+H+	66.98	19.8	0.004
V−H−/V+H−	59.65	17.5	0.004
V−H−/V−H+	39.27	19.7	0.19
V−H+//V+H+	27.71	20.5	0.53
V+H−/V+H+	7.33	16.6	0.97
V−H+/V+H−	20.38	17.7	0.66

Both the V+H+ and the V+H− conditions yielded significantly faster reaction times than the V−H− condition. In contrast, no significant differences in the RTs have been found for the V−H+ condition, neither in comparison with the V+H− condition nor with the V−H− condition.

In the effect-coded model, the same random effects improved the model fit and were added to the final model. The resulting parameter estimates revealed a significant influence of both VL and harmony, but not of their interaction, on RT. The estimated intercept was 733, −60 for good VL (P = 0.0002) and −39 (P = 0.01) for good harmony (Table 2).

**Table 2:** Output for the fixed effects from the generalised mixed effects model with VL and harmony as effect-coded variables in the fixed effect.

	Estimate	Standard error	T value	P-value
Intercept	733.2	17.5	41.9	<0.0001
VL	−59.7	15.9	−3.7	0.0002
Harmony	−39.3	15.7	−2.5	0.01
VL x Harmony	31.9	17.8	1.8	0.07

## Discussion and Conclusion

The results of the present study challenge the widely held view that harmony is the main component in guiding listener expectations in Western polyphonic tonal music and provide evidence that VL is another crucial component, one that may be even more important than harmony. The findings are consistent with previous studies according to which music theoretically expected endings of a sequence yield faster reaction times (RTs)^11,12^. Harmony had a significant influence on RTs, which is in accordance with previous studies. In addition, VL also had a significant influence with an estimated difference that was even larger than that of harmony. Moreover, in pairwise comparisons the V+H+ and the V+H− conditions both yielded significantly faster RTs than V−H−.

In contrast, the V−H+ condition did not show significant differences in RTs to the V−H− condition. In addition, the difference between the conditions of main interest, V+H− vs V−H+, did not reach the level of significance either. This might be explained by the fact that the targets were designed in such a way that neither VL nor harmony were extremely dysfunctional but only moderately *poor* in the respective conditions (see Fig. 1). Nevertheless, the estimated difference in RTs was larger for *good* vs *poor* VL (−59.7) than for *good* vs *poor* harmony (−39.3). This might reflect the historical priority of intervallic VL over root-based harmony.

In the normal timbre, incorrect answers occurred twice as often in the V−H− condition compared to all other conditions. Apparently, participants were more likely to confuse a V−H− ending with a change in timbre. A potential explanation for this effect could be the fact that in both cases the frequency spectra change abruptly, due to leaping voices for the V−H− condition and due to a change in timbre, respectively. However, the overall amount of incorrect answers was very low, indicating that the task was easy enough also for non-musicians. We found that musicality (general sophistication from the Gold-MSI) did not have any notable effect on the responses, which is largely consistent with the findings in previous harmonic priming studies^4,6,13^. Therefore, the structural properties of the stimuli themselves and how they interact with mechanisms of expectancy need to be addressed in more detail.

Following Huron^35^, expectation can be defined as featuring a when- and what-component. Since the rhythm remained constant throughout each stimulus, only the what-component is relevant here. The what-component can be characterised by differing degrees of abstraction, ranging from concrete pitches to pitch classes or, even more generally, to textures of differing degrees of spacing and density. Against this background, one explanation for the slight dominance of VL over harmony may be that harmonic analysis requires a listener to engage in a process of root inference^36^. It may be argued that root inference is supposedly more time-consuming than evaluating the local intervallic relationship between both spatially and temporally adjacent notes. Situations featuring *good* VL (V+H−) generally produce compact, regular and predictable textures. If salient outer-voices (especially the soprano) are treated in a predictable fashion governed by minimal VL, this may compensate for local harmonic deviations. In contrast, VL deviations (as in V−H+) often result in skips and sudden registral changes, which seem to hinder fast processing. These surface changes had a higher impact on RT than harmonic deviations in the V+H− condition.

As the stimuli were composed by a music theory expert, a subjective component in the goodness of VL and harmony cannot be ruled out, despite intersubjective validations. A precise formalisation of the rules for both VL and harmony and their interaction (see Musical Stimuli) would enable an automated stimulus generation and further reduce the subjective component involved in assessing the goodness of the stimuli in relation to VL and harmony.

While our study adopts a more complex notion of VL than previous empirical work (e.g.^13^), it does not allow to weigh the specific impact of the individual VL components, due to the limited number of stimuli. Using a larger amount of stimuli, follow-up studies might examine the specific impact of different features in both the VL and the harmonic domains for predicting the perceived well-formedness of a polyphonic sequence. An automated stimulus generation would not only help in composing stimuli that follow well-defined criteria; it would also allow to make use of larger and more diverse sets of stimuli. Therefore, based on the present study, there are two main avenues for future research: (1) the automated generation of stimuli based on a thorough formalisation of rules for both VL and harmony and (2) the subsequent empirical investigation in cognitive experiments, which would then allow a more detailed analysis of the influence of the different components within the two domains on expectation formation.

To summarise, the findings reported here largely conform to prior music theoretical intuitions, providing evidence in favour of our hypothesis according to which voice leading and harmony are closely intertwined and hence jointly work together in guiding listener expectations. Also, there is evidence in favour of the hypothesis that VL has a slightly stronger effect on expectancy than harmony. This would imply that the well-formedness of VL can compensate for harmonic deficiencies.

## Supplementary information

Supplementary Information.

Supplementary Information2.

Supplementary Information3.

## Data Availability

The datasets generated during and/or analysed during the current study are available from the corresponding author on request.
