# Supplementary material for: The Impact of Voice Leading and Harmony on Musical Expectancy
Source: Sci Rep. 2020 Apr 3;10:5933. doi: 10.1038/s41598-020-61645-4 (PMC7125180; doi:10.1038/s41598-020-61645-4)
Supplement: Supplementary file 2 — Supplementary Information2. [file 41598_2020_61645_MOESM2_ESM.pdf]

The Impact of Voice Leading and Harmony on Musical Expectancy

Leona Wall[1]*, Robert Lieck[1]**, Markus Neuwirth[1], and Martin Rohrmeier[1]

[1] Digital and Cognitive Musicology Laboratory, École Polytechnique Fédérale de Lausanne, 1015 Lausanne, Switzerland

*research@leona-wall.com
**research@robert-lieck.com

Supplementary Material

Stimuli

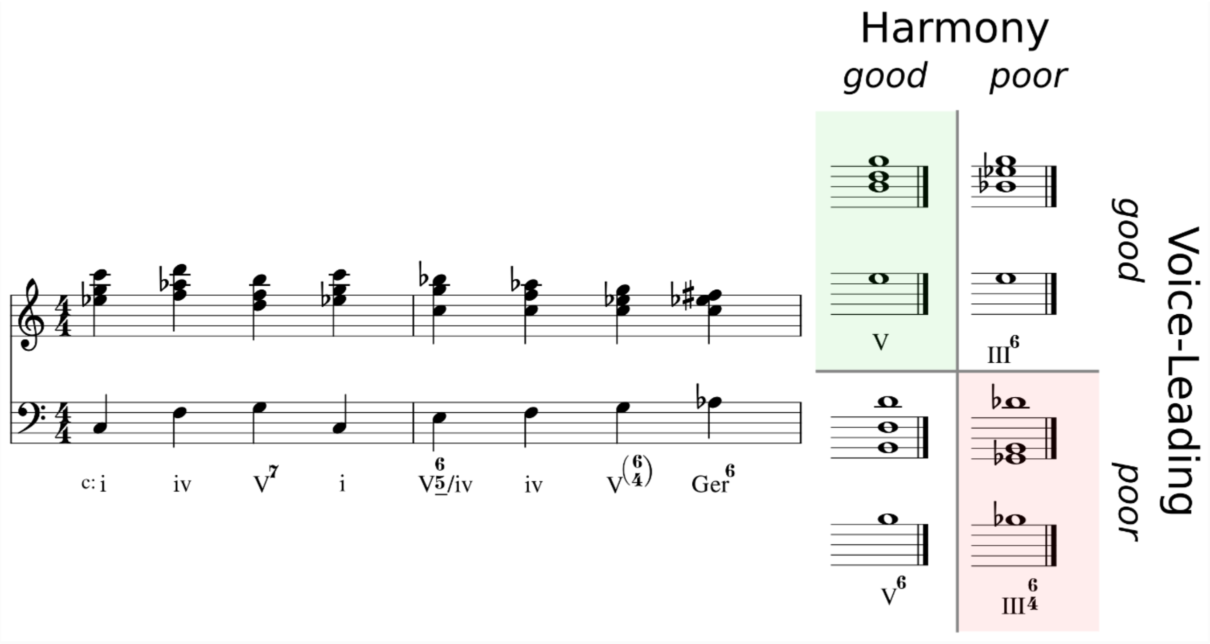

Supplementary Figure F1: Stimulus 1

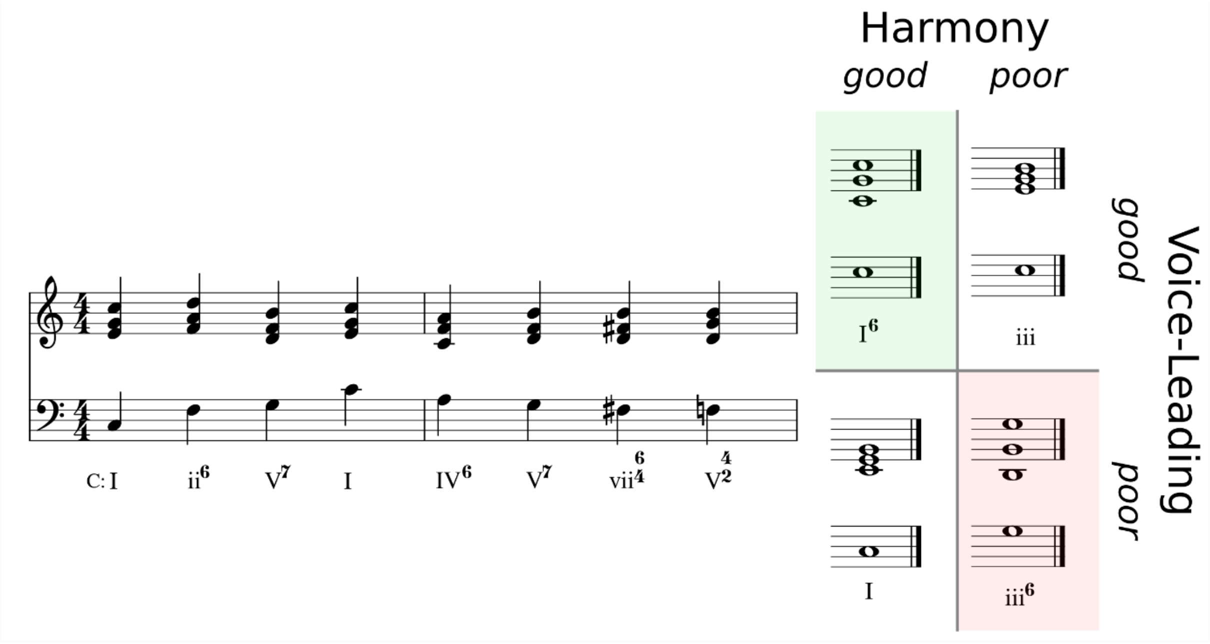

Supplementary Figure F2: Stimulus 2

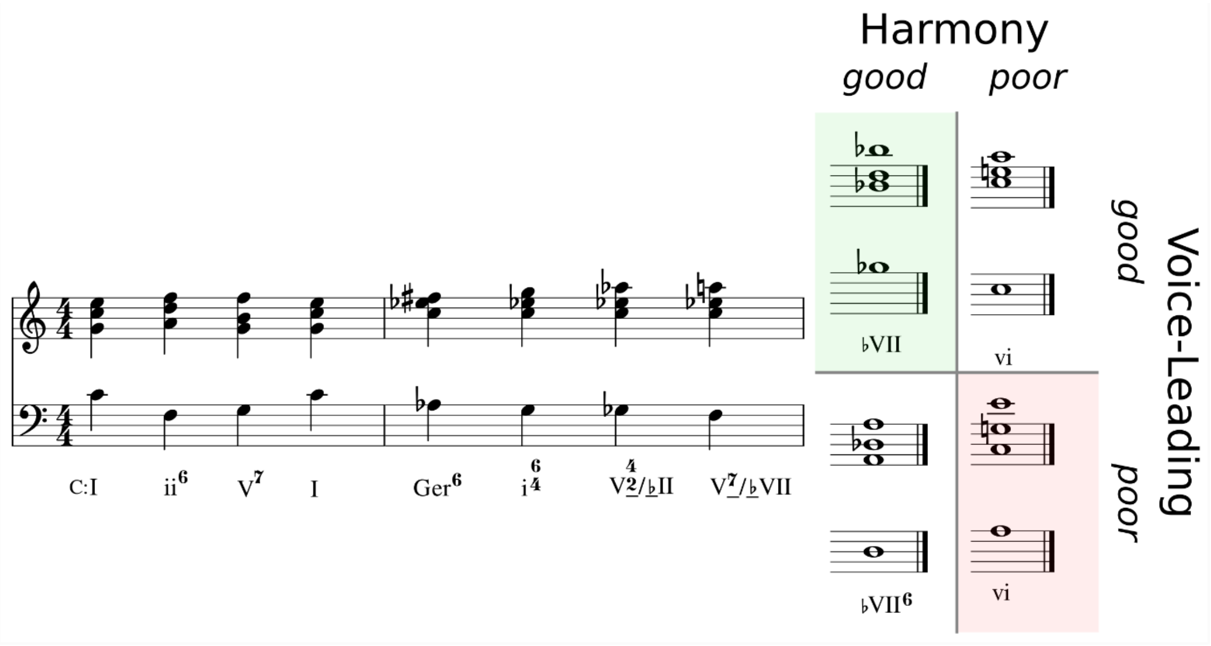

Supplementary Figure F3: Stimulus 3

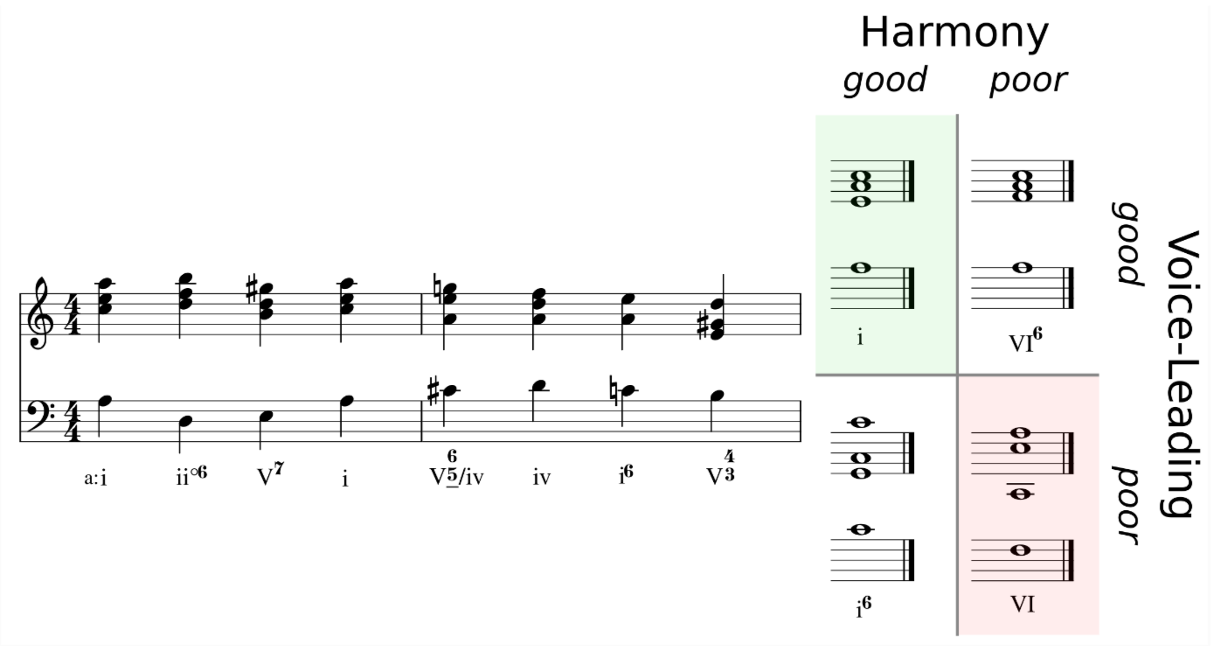

Supplementary Figure F4: Stimulus 4

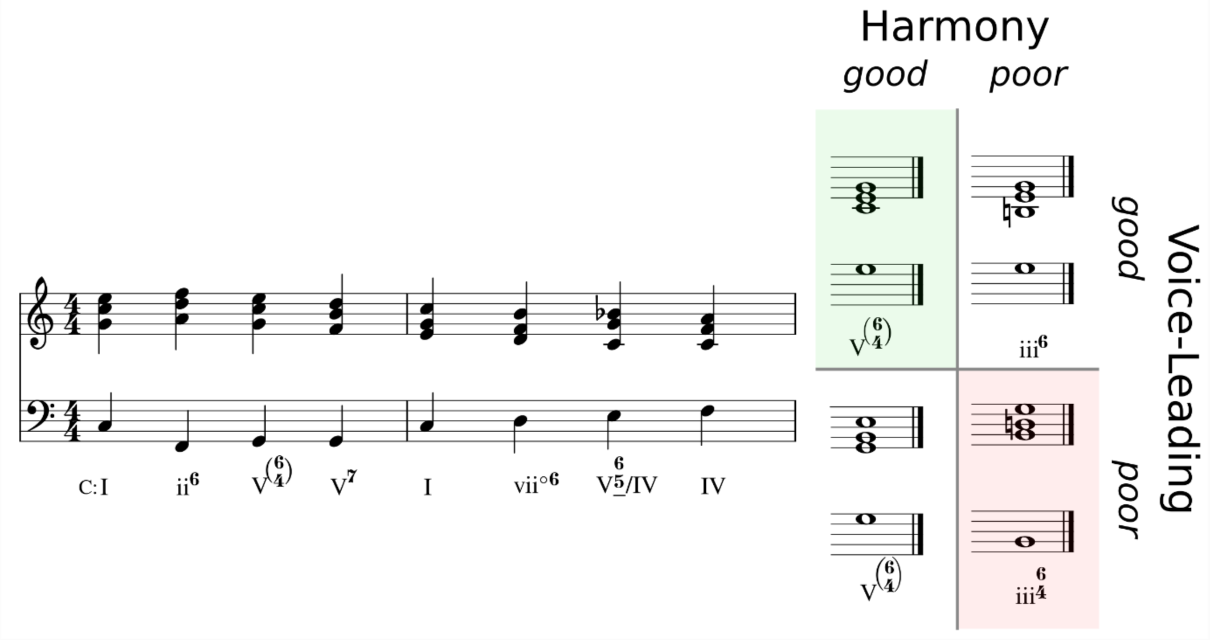

Supplementary Figure F5: Stimulus 5

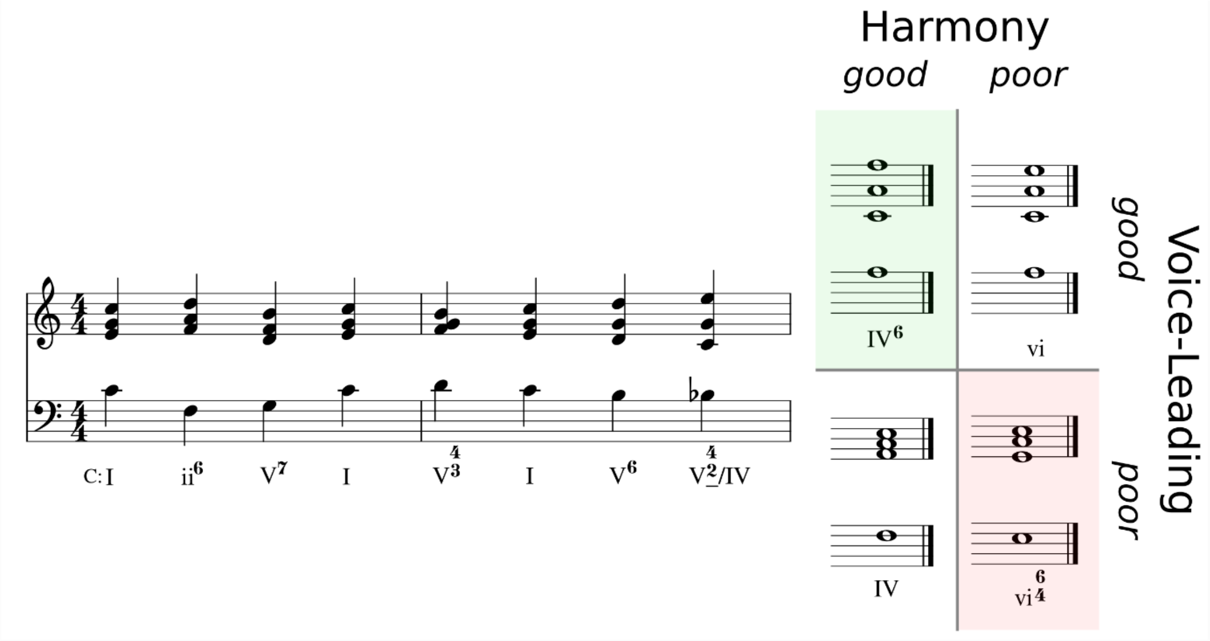

Supplementary Figure F6: Stimulus 6

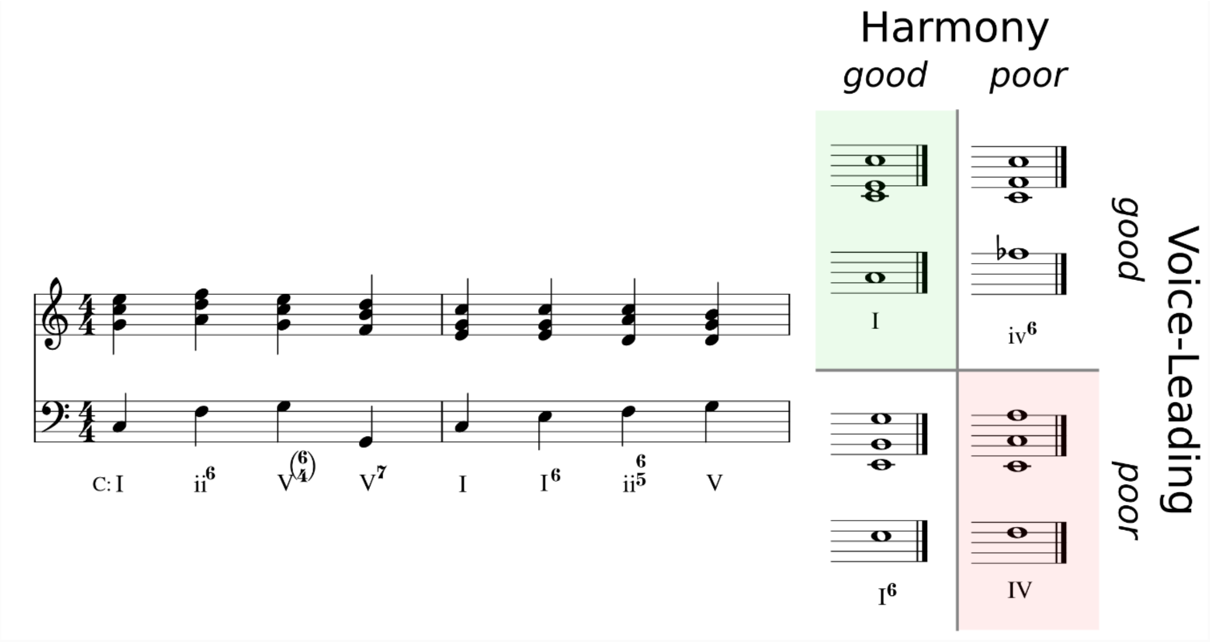

Supplementary Figure F7: Stimulus

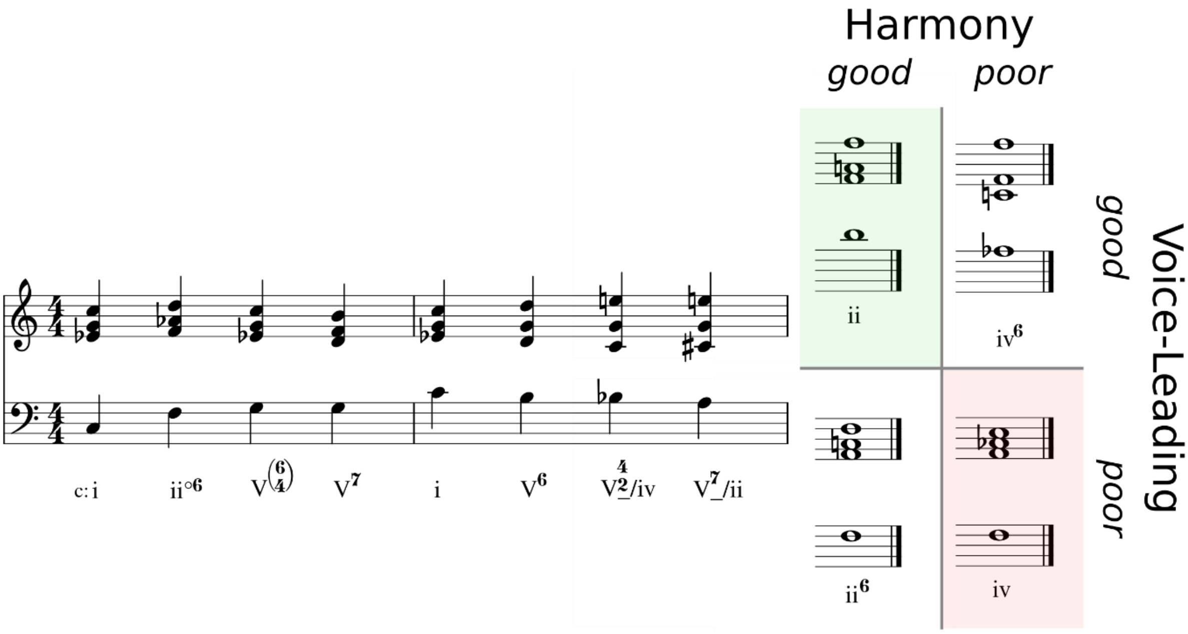

Supplementary Figure F8: Stimulus 8

Internal rating of the stimuli

To ensure the functional quality of the stimuli, a lab-internal evaluation on the audio files was performed prior to the experiment. The stimuli were presented in random order to individuals from the laboratory with sufficient musical expertise. The instructions were to rate the stimuli regarding their quality in voice leading or harmony on a scale from 0 (catastrophic) to 10 (very good) with 0.5 steps. Every stimulus was played twice, but not consecutively, so that voice leading and harmony were rated independently. The individuals were familiar with the purpose of the study but not involved in the design of the stimuli.

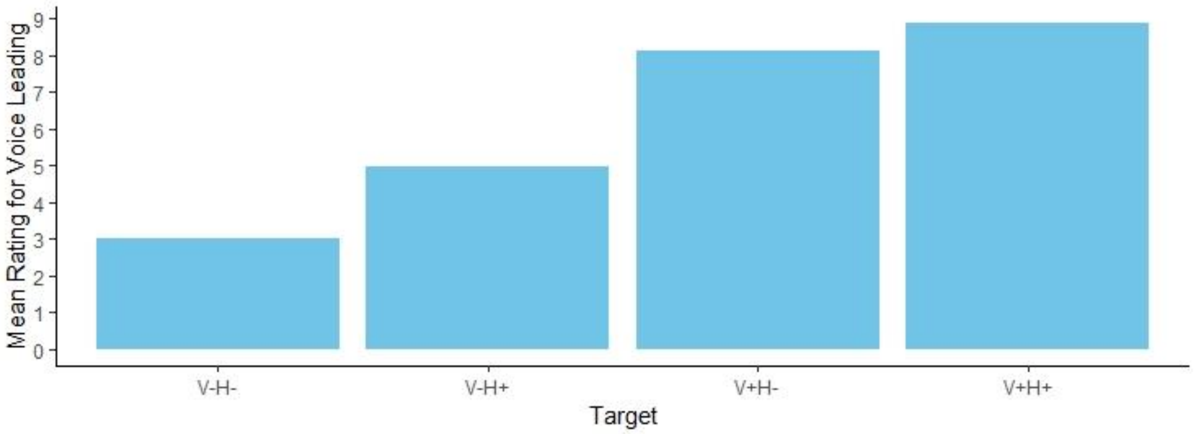

Supplementary Figure F9: VL ratings for the stimuli: 0 is highly deficient VL, 10 is perfect.

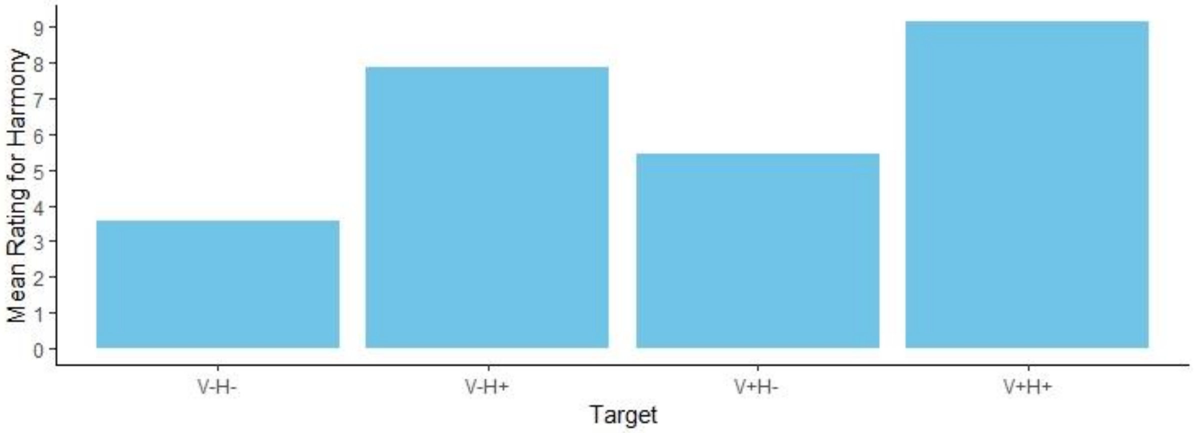

Supplementary Figure F10: Harmony ratings for the stimuli: 0 is highly deficient harmony, 10 is perfect.

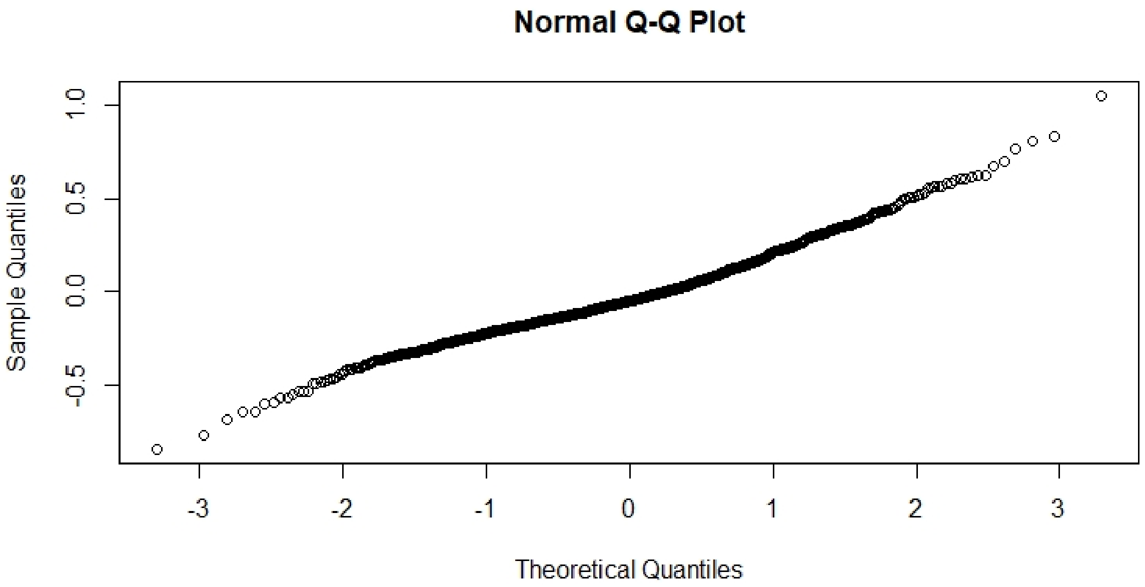

Supplementary Figure F11: Q-Q Plot of the residuals of the final generalised mixed effects model.

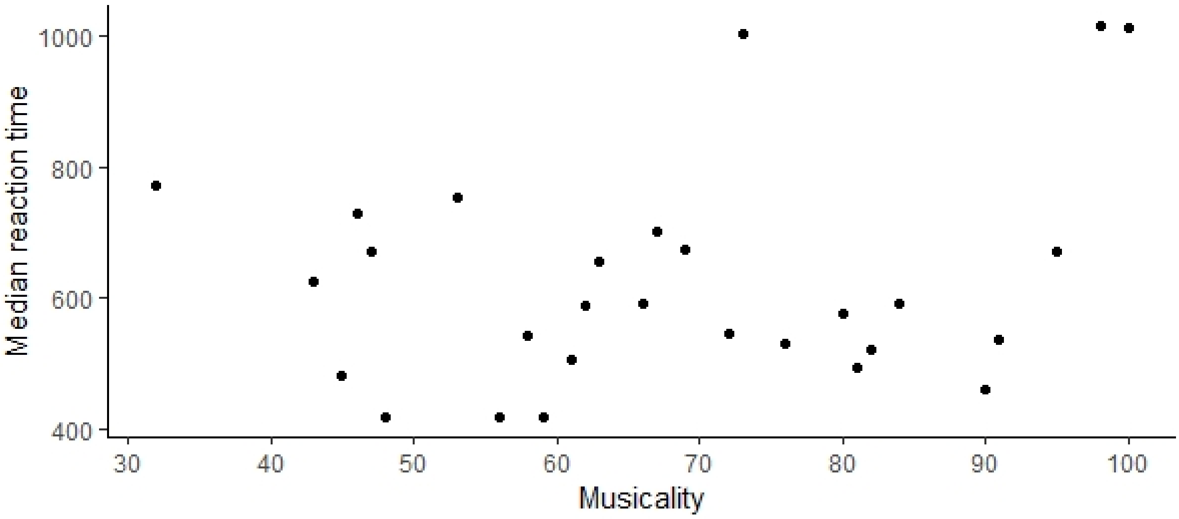

Supplementary Figure F12: Median reaction time per musicality.

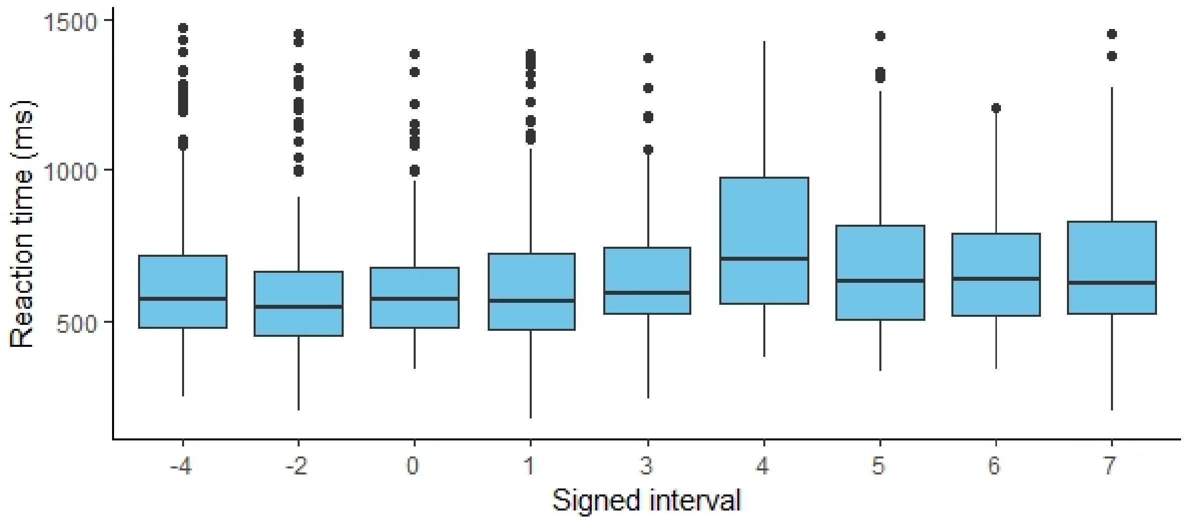

Supplementary Figure F13: Boxplots of reaction time for the different final melodic intervals of the target compared to the last chord of the prime sequence.
